# The effects of attention capacity on dynamic balance control following concussion

**DOI:** 10.1186/1743-0003-8-8

**Published:** 2011-02-03

**Authors:** Robert D Catena, Paul van Donkelaar, Li-Shan Chou

**Affiliations:** 1 Motion Analysis Laboratory, Department of Human Physiology, University of Oregon, 97403 Eugene, Oregon, USA

## Abstract

The purpose of this study was to examine how individuals modulate attention in a gait/cognition dual task during a 4-week period following a concussion. Ten individuals suffering from a grade 2 concussion and 10 matched controls performed a single task of level walking, a seated auditory Stroop task and a simultaneous auditory Stroop and walking task. Reaction time and accuracy were measured from the Stroop task. Dynamic balance control during gait was measured by the interaction (displacement and velocity) between the center of mass (CoM) and center of pressure (CoP) in the coronal and sagittal planes. Concussed individuals shifted from conservative control of balance (shorter separation between CoM and CoP) immediately after injury to normal balance control over 28 days post-injury. Immediately after injury, correlations analyses using each subject on each testing day as a data point showed that there was a spectrum of deficient performance among concussed individuals on the first testing day. Within a testing session, deficiencies in reaction time of processing involved in the Stroop task were commonly seen with reduce dynamic balance control. However, the prioritization was not always towards the same task between trials. There were no correlations in the control group. Information provided in this study would enhance our understanding of the interaction between attention and gait following concussion.

## 1. Background

Previous research has demonstrated the use of attention to modulate balance control and cognition when the two are used/needed simultaneously [1-7]. The prevailing belief is that there is a tradeoff between tasks as the two tasks combined become too challenging [8,9]. Although divided attention has been shown to cause gait imbalance after concussion [5], the interaction between cognition and gait has not been specifically outlined following concussion. How this interaction presents itself, as either a prioritization (a shift of attention towards one task at the cost of the other) of a particular task or an overall reduction in performance in both tasks, and how it changes over time after brain injury, has not been examined.

While reports of professional athletes suffering long-term quality of life issues have become more prevalent in recent years, scientific research has struggled with the possibility that long-term effects from concussion might exist [10-12]. Motor deficits, either short- or long-term, following concussion have typically gone relatively unstudied compared to other neurological deficits (e.g. Parkinson's or cerebral palsy). Of particular interest has been how attention deficits following a concussion alter motor performance. Concussed individuals with a reduced ability to spatially orient attention had a tendency for lower clearances while stepping over obstacle and more obstacle contacts during gait [13]. The interaction between deficits in spatial orientation of attention and obstacle clearance decreased by 6 days after the concussion, and was no longer present at 14 days. These results implied that there exists a particularly strong interaction between attention and motor control when individuals are suffering the full effects of the concussion, but as the injury is transient so is the interaction. Recently, tests of dynamic balance control in conjunction with a secondary cognitive task have been proposed as an alternative method for assessing the resolution of deficits following concussion [1,2,14]. However, the tasks that were used in previous studies were not able to discern specifically how attention modulates the interaction between dynamic imbalance and cognitive deficits following a concussion.

The purpose of this experiment was to analyze attention during the interaction between cognition, through a Stroop reaction time task, and dynamic balance control following a concussion. An interaction was hypothesized based on well-understood data in healthy individuals [15,16], but how exactly this will present itself following a concussion is unknown. This research will indicate how concussed patients resolve issues with performing a dual-task involving an executive functioning measure and gait. Information from this study will also help us understand the influence of attention capacity deficits in a limited-capacity system since previous studies with similarly rigorous dual tasks have been dually examined, but not dually quantified.

## 2. Methods

### 2.1 Subjects

Twenty young adults participated in this study. Subjects were divided into two groups: ten subjects suffering from concussion or mild traumatic brain injury (mTBI) and ten controls without injury (Cont). The experimental protocol was approved by the Institutional Review Board at the University of Oregon. Written and verbal instructions of testing procedures were provided, and written consent was obtained from each subject prior to testing.

Participants suffering from a grade 2 concussion (5 females/5 males; age = 21.0 ± 3.1 years; body mass = 71.7 ± 10.5 kg; height = 173.6 ± 11.5 cm) were initially recruited for testing within two days following the injury after being identified and diagnosed by certified athletic trainers and/or attending medical doctors in the university intercollegiate athletic program or the student health center. Subsequent testing occurred 6, 14 and 28 days after the injury. The severity of the injury was categorized by the attending certified athletic trainers and/or medical doctors in accordance with the definitions originated by the American Academy of Neurology [17]. During the initial medical diagnosis, a grade of "2" was assigned if the participant remained disoriented for greater than 15 minutes, but did not lose consciousness for any period of time. All participants were re-evaluated by a single researcher about their symptoms and diagnosis when arriving for their first testing to confirm that they did suffer a grade 2 concussion. All concussion participants were asked to fill out a questionnaire of symptoms, and a range of symptoms and severities were recorded without a clear pattern or trend. Age-, gender-, athletic ability (sport)-, education level-, height- and body mass-matched (age = 20.7 ± 4.1 years; body mass = 72.6 ± 10.5 kg; height = 172.7 ± 11.6 cm) control participants were recruited and tested for the same intervals. Injuries/disorders that prevented normal gait (e.g. exhaustion, sprains, and ataxia) were exclusion criteria applied to all subjects and common symptoms of concussion were exclusion criteria applied to control individuals.

### 2.2 Experimental protocol

Participants performed: (1) single-task level walking, (2) a seated auditory Stroop task and (3) walking with an auditory Stroop task. The auditory Stroop task required the participant to listen to a computer presented word ("high" or "low") that was presented in either a high or low pitch. The objective of the subject was to always declare the pitch of the word while ignoring the word itself. Congruent (where the pitch matches the word) and incongruent (where the pitch doesn't match the word) conditions were examined separately to measure the Stroop effect, and analyzed together to analyze attentional capacity. Blocks of four seated Stroop trials were performed before and after walking trials.

Walking trials were performed in blocks of eight trials for single- and dual-task. The order of walking trials was randomized for each subject and each day. Several minutes of rest were provided between blocks. Walking was performed along a 10 m walkway at a self-selected pace. During Stroop walking, a single stimulus was presented at the beginning of the analyzed motion data of one complete stride. Subjects were informed about the impending task at the beginning of each block of trials.

### 2.3 Experimental apparatus

Twenty-nine retro-reflective markers, modified from the Helen Hayes marker set [14], were placed on anatomical landmarks. Three dimensional marker trajectories were collected with an eight camera motion tracking system (Motion Analysis Corp., Santa Rosa, CA) at a sampling frequency of 60 Hz. Ground reaction forces and moments in three orthogonal directions were collected at a sampling rate of 960 Hz with two in-series strain gauge force plates (Advanced Mechanical Technologies Inc. Watertown, MA) flush with the top surface of the floor in the center of the walkway. Stroop stimuli were presented in random order using SuperLab Pro (Cedrus Corp. San Pedro, CA). Responses were recorded with Motion Analysis software.

### 2.4 Data Processing

**Figure 1:**
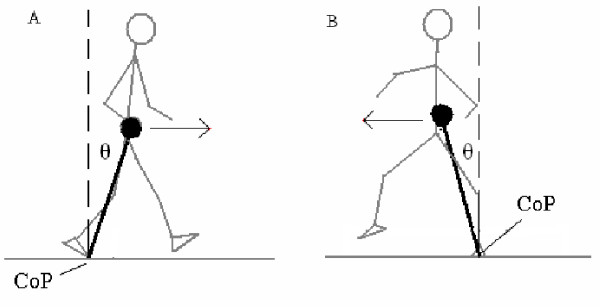
**Center of mass (as the head of the inverted pendulum) to center of pressure inclination angles in the (A) sagittal and (B) frontal planes**.

Besides recording response accuracy, we also analyzed reaction times during the Stroop task with a radio-telemetric microphone. Voice recordings were collected at 960 Hz. Visual inspections by a single examiner determined the onset of all responses. For the gait analysis, marker trajectories were filtered with a low-pass fourth order Butterworth filter at a cutoff frequency of 8 Hz. Marker position data were used to locate the segmental centers of mass (CoM) of a thirteen-link model based on Dempster's [18] anthropometric data. A weighted sum method was used to calculate the whole body CoM from segmental CoMs during each time point. CoM data were analyzed between the first heel strike on to the first force plate to the next heel strike of the same foot. CoM velocities were estimated with the use of Woltring's generalized cross-validated spline algorithm [19]. Center of pressure (CoP) was calculated using the ground reaction forces/moments measured with the two force plates. The CoP data were then time-synchronized with the motion data. During the double stance phase, a resultant CoP was calculated for both feet using the CoP and vertical ground reaction force from each foot. Inclination angles were calculated as the angle from the CoM, down to the CoP and back up to the vertical (Figure 1). Peak inclination angles in the sagittal and coronal plane were identified during a gait cycle. The posterior and anterior peak angles were then summed to find a sagittal plane angular range of motion (SR). The medial peak angle from the left foot was summed to that of the right foot to find a frontal plane angular range of motion (FR). Peak CoM velocities in both the sagittal and frontal plane (SV, FV) were also calculated. These balance control variables have been used previously to identify balance control deficits in individuals with a history of falling [20].

### 2.5 Statistical analyses

A three-way mixed model analysis of variance with repeated measures (alpha = 0.05) was conducted on each variable using SPSS v.12 (SPSS Inc., Chicago, IL). Testing day and task were within-subject factors and group was a between-subject factor. Previously described balance control and Stroop variables were dependant variables of interest. The appropriate assumptions for both within and between subjects ANOVA were considered. Pairwise comparisons in the three way interaction were analyzed with adjustments for multiple comparisons (alpha = 0.05/8 = 0.00625), while other pairwise comparisons were analyzed with Least Significant Difference adjustments for multiple comparisons.

To analyze how each group altered one task performance with respect to the other in the dual-task situation we calculated correlations between the means of balance variables and Stroop reactions times with linear regressions for each group and day (alpha = 0.05). To analyze how each individual altered one task performance with respect to other in the dual-task situation we calculated correlation coefficients for each individual and then conducted t-tests on individual correlation coefficients between groups (alpha = 0.05).

## 3. Results

### 3.1 Group comparisons of Stroop and balance performance

Analyses of Stroop reaction time separated by group, testing day and task specifics (congruent vs. incongruent Stroop task or motor task) indicated no statistically significant differences during seated (*p *= 0.556) or walking trials (*p *= 0.735). The average reaction time varied between 777 ms and 1019 ms depending on the group, testing day and task specifics, with no clear trends. There were also no statistical differences in response accuracy as there were never any incorrect responses.

**Figure 2:**
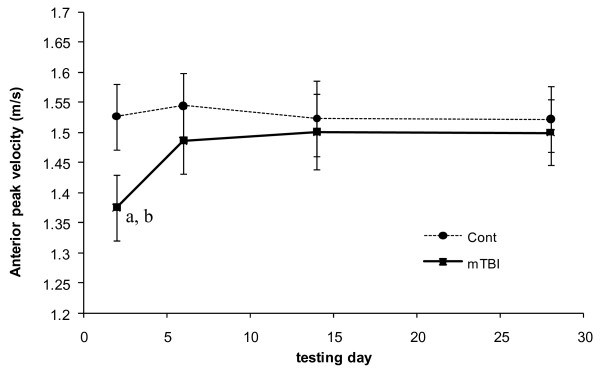
**Anterior peak velocity of center of mass over 28 days of testing for each group**. Controls are represented by the dashed line. Concussed are represented by the solid line. Standard error bars are presented. (a) Indicates a trend of statistical difference (p = 0.064) between the two groups on a particular day. (b) Indicates statistical difference (p = 0.001) from all other days in a particular group.

**Figure 3:**
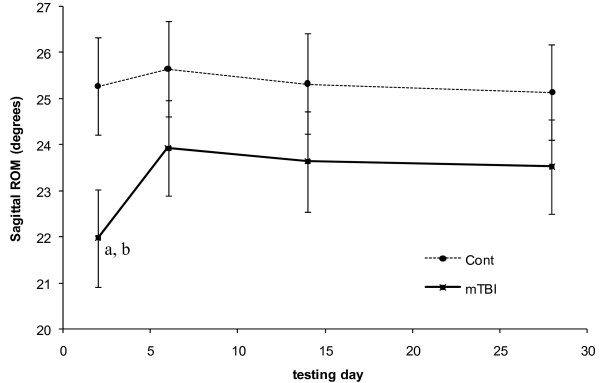
**Sagittal plane angular range of motion of the center of mass with respect to the center of pressure over 28 days of testing**. Controls are represented by the dashed line. Concussed are represented by the solid line. Standard error bars are presented. (a) Indicates statistical difference (p = 0.041) between the two groups on a particular day. (b) Indicates statistical (p = 0.001) from all other days in a particular group.

Both peak anterior CoM velocity and angular range of motion in the sagittal plane indicated group*day interactions (*p *= 0.004 and *p *= 0.015, respectively) and task differences (*p *= 0.003 and *p *= 0.021, respectively). Group*day interactions indicated that only concussed individuals walked with significantly slower sagittal CoM motion (Figure 2) and allowed less sagittal plane CoM-CoP angular separation (Figure 3) on the first testing day compared to all other testing days. Both groups walked with significantly slower peak velocities and allowed significantly less sagittal plane CoM-CoP separation during single task walking compared to Stroop walking.

**Figure 4:**
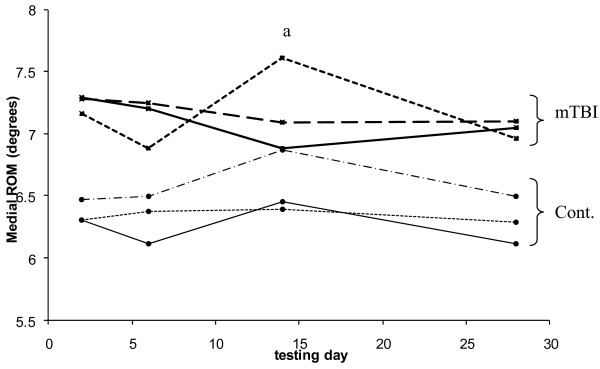
**Medial angular range of motion of center of mass with respect to center of pressure over 28 days of testing**. Concussed subjects are represented by "mTBI" to the right of the graph and control subjects are represented by "Cont." Solid lines represent single task walking, short-dashed lines represent Stroop walking during congruent task presentation and long-dashed lines represent Stroop walking during incongruent task presentation. (a) Indicates statistical difference (p < 0.00625) between the two groups for congruent Stroop walking on day 14.

Analysis of variance on frontal plane angular range of motion of the CoM (Figure 4) indicated a group*day*task interaction (*p *= 0.003). The concussed group in general tended to have more frontal plane motion than controls during gait, but specifically showed increased motion during congruent Stroop walking on the day 14 testing (*p *= 0.006). This difference was not due to the fact that control individuals changed, as they did not have any statistical difference between days, but because concussed individuals had increased frontal plane motion during congruent Stroop walking on day 14 compared to day 7 (*p *= 0.002) and day 28 (*p *< 0.001) post-injury. Frontal plane motion in the concussed individuals during congruent Stroop walking on the day 14 testing was also significantly greater than single-task walking on the same day (*p *= 0.003). There were no statistically significant differences in peak medial velocity of the CoM.

### 3.2 Correlation analyses of Stroop and balance at the group and individual level

**Figure 5:**
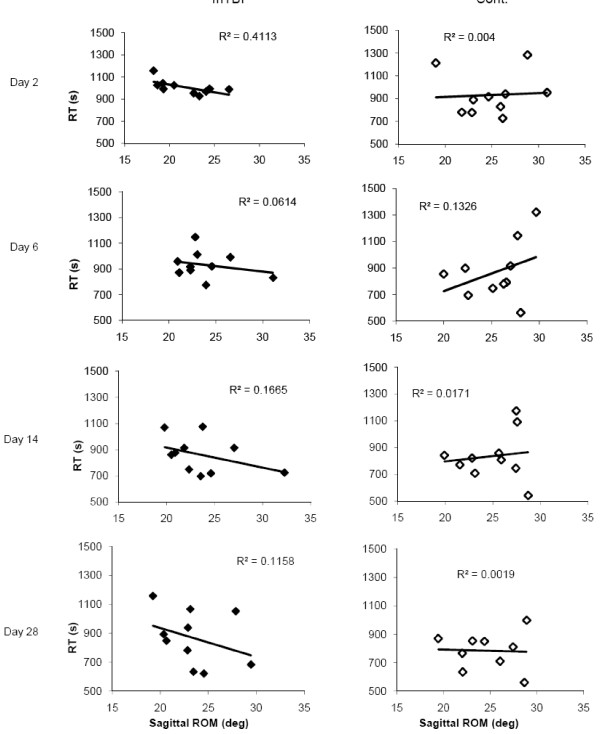
**Correlation between sagittal plane angular range of motion of the CoM with respect to the CoP and Stroop reaction time for concussed and control individuals on each testing day**. Each point represents the mean performance of a single individual.

Control individuals demonstrated no correlation between sagittal plane motion and Stroop performance. On the other hand, concussed individuals showed significant moderate correlations between sagittal plane motion and Stroop performance during gait 48 hours after injury (R^2 ^= .411, *p *= 0.046), which reduced to non-significant levels on the subsequent testing days. The relationship showed that concussed individuals who displayed shorter sagittal plane CoM-CoP angles had longer reaction times in the Stroop task (Figure 5).

When specifically looking within each individual we found that seven of the ten concussed individuals displayed a positive correlation between Stroop reaction time deficits and sagittal angular range of motion on a trial-by-trial basis 48 hours after injury, indicating increased deficits in one task may correlate with increase deficits in the other task. The correlation coefficients for concussed individuals were statistically different than control individuals who displayed no correlation (n = 4) or the opposite correlation (n = 5) within most individuals (*p *= 0.048). There were no statistical differences between the correlation coefficients of each group on subsequent testing days. By 6 and 14 days post-concussion only one individual was still exhibiting this shift in prioritization between trials. By 28 days, he performed similar to controls.

## 4. Discussion

This research examined attention prioritization (a shift of attention towards one task at the cost of the other) between dynamic balance control and cognitive performance. Information was used to estimate if and how attention capacity deficits and/or executive functioning following concussion affect dual task performance.

### 4.1 Executive functioning

Executive function is a higher level manager of other cognitive processes. One of its subtasks is conflict resolution, which allows us to ignore conflicting information to choose an appropriate response. It has been suggested that balance deficits could be due to an interaction with executive functioning deficits in certain pathological groups, including traumatic brain injury [21]. This was not indicated in the current study because there were no clear differences in the effects of congruency on balance control. The single possible indication of an executive function interaction with dynamic balance control following concussion occurred at the day 14 testing. Concussed individuals performing a congruent Stroop task had more coronal plane motion than when they were performing single task walking. Incongruent Stroop walking did not result in the same increased coronal plane motion. However, since there was no statistical difference between congruency conditions then this is only a speculative difference until further studies can shed more light on this particular trend. We have no definitive explanation for day 14 findings; however a reoccurrence of balance deficits has been previously found and explained by a return to sport and activity too soon [5].

### 4.2 Attention Capacity

Cognition - gait balance control interactions were much more obvious in this study as demonstrated by the fact that concussed individuals who had slower reaction times overall in the Stroop task also used a more cautious gait strategy (as indicated by decreased CoM-CoP inclination). A similar conservative gait strategy has been found in mild [1,2,4,5] and more severe [22,23] brain-injured individuals. These changes are thought to be an effort to reduce the CoM forward momentum during gait progression [24,25] and may indicate a degraded ability to maintain gait stability in individuals suffering from a concussion. While the reduction serves the mechanical purpose of making balance in the sagittal plane easier to maintain, it still remains unclear if these are inherent or conscious adjustments made by the individual. The finding of deficits in reaction time in concert with the conservative gait strategy in certain individuals points to concussion being a broad reduction in performance rather than isolated to specific areas. Similar to the transient nature of concussion, these deficiencies in dual-task performance gradually subsided.

The spread in performances indicates that within the group of concussed individuals there are individuals that may be more impaired than others. Those that possibly suffered more impairment appeared to have a general cognitive deficit along with an average slowing of motor performance in the dual-task situation compared to those that may have more mild injury or may at the time be completely recovered. This is supported by findings that severely brain injured individuals performed poorly in postural control while at the same time committing more arithmetic errors in a dual-task paradigm [3]. This information, from a group of individuals assigned the same concussion grade, shows the importance of diagnosing a concussion beyond simple grading scales that categorize patients of varying severity into the same group. This interaction was only statistically evident in the first testing session, after which it gradually decreased. Presumably, this is due to the recovery from the effects of concussion that individuals suffered soon after the injury. The fact that first testing session correlations were not near perfect correlations indicates that using balance control as the sole indicator for determining an athlete's readiness to resume competitive activity after injury is not recommended.

In the analysis of individual concussed participants we looked at trends in balance/cognition interactions between trials for each individual. We then used that information to distinguish between concussed and control individuals. Within each concussed individual (between trials) on the first testing day we found that most individuals had a trade-off, or prioritization, in performance of tasks in the dual-task situation. For certain trials they had quicker reaction times with more cautious sagittal plane motion and for other trials they had slower reaction times with less cautious gait performance. This interaction was not seen in control individuals in the first testing session and in three concussed individuals it was not present. We can only explain a lack of prioritization shifts between trials not present in three concussed individuals by a lack of deficits in control of prioritization (similar to controls) for these individuals. This may be due to severity or recovery rates differing among individuals. In subsequent testing sessions there were no statistical differences between how concussed and control individuals varied secondary task performance with walking strategy.

This interaction and resultant trade-off has not been previously examined in concussed individuals during a dynamic motor task, but it is similar to what healthy individuals have displayed in coordination/reaction time tasks [15,16]. The fact that we did not observe this trade-off in control individuals for the particular paradigm used in this study is promising in that it might indicate that the dual-task scenario is particularly sensitive to the effects of concussions. Trade-offs in task performances seem to be a result of limited attentional resources indicated in the group correlations, similar to previous findings [15]. As attention resources diminish, two tasks that were was once simply performed simultaneously without any degraded performance (as in controls) now compete for attention to be accomplished at all. The fact that these interactions are apparent only immediately after concussion are particularly noteworthy since our previous research has shown dual-task balance deficits immediately after concussion [1,2,4,5], but slowly subsiding thereafter [5].

Interestingly, the prioritization to a particular task that a concussed individual showed in one trial was not necessarily the same that they would use for the next trial (i.e. prioritization shifted between tasks). There were no clear trends in how the prioritization shifted within a testing session, but we would recommend that this be investigated further with an increased sample of trials. Individuals have previously exhibited a prioritization of a balance control in a dual task situation [7]. This "posture first hypothesis" indicates that individuals would prioritize control of balance before another safer task [26]. The fact that concussed individuals shifted prioritization between tasks during separate trials would seem to indicate they did not fully comprehend their poor control over balance and/or were unable to appropriate focus attention on the walking task. As previously stated, current information on the prioritization of attention following concussions is lacking. This information would be helpful in understanding the interaction between cognition and motor control. This current study was unable to detect trends in prioritization following concussion, which actually is useful information given the frequently exhibited prioritization on balance control in healthy individuals. This information may prove useful in future developments of secondary measures of a persisting brain injury.

### 4.3 Limitations

There were limitations sources in this study beyond those already described in the discussion. Baseline information would have been a more reliable source for comparison of deficits as a result of concussion. The inclusion of a motor task makes baseline information very difficult to obtain. Making group comparisons with control individuals was a major source of variability. Sample size is an obvious concern with our strict exclusion criteria. A lack of statistical power may have made certain findings appear to lack a statistical significance (e.g. Stroop reaction times) when an increased sample size would have found differently. Furthermore, a limited number of Stroop trials per block increased variability in reaction times within individuals. It seemed that an auditory Stroop task was more variable than a visual Stroop reaction time usually employed. When used as a secondary task in a dual-task scenario, the auditory Stroop task can provide the desired rigor, but unfortunately also requires additional trials to make more concrete conclusions in its performance when analyzed with an ANOVA test.

## 5. Conclusions

Even though attention deficits have been previously shown to be related to balance control after concussion, there were few indications that dynamic balance control was affected by executive function deficits. However, it should be noted that deficits were not even noted in the seated trials, possibly indicating the inappropriateness of the task for measuring executive functioning following concussion. On the other hand, changes in gait due to concussion were evident in individuals that also had slower reaction times in the secondary Stroop task. While analyses of the concussed group as a whole indicates that those that have deficits in one task also suffer deficits in the other, the analyses of inter-trial task performances within the concussed group showed most individuals used a trade-off in task performances while controls did not show this trend. This only occurred on the first testing day. This information points to attentional capacity deficits as a possible contributor to gait abnormalities following a concussion.

## Competing interests

The authors declare that they have no competing interests.

## Authors' contributions

All authors have made substantial contributions to study conception and design. RDC took the primary responsibility in data collection, processing and analysis, with the assistance from LSC and PVD. RDC and LCS performed interpretation on findings and drafted the manuscript. All authors read, provided comments and suggestions, and approved the manuscript.
